# Supplementary material for: Brainprints: identifying individuals from magnetoencephalograms
Source: Commun Biol. 2022 Aug 22;5:852. doi: 10.1038/s42003-022-03727-9 (PMC9395342; doi:10.1038/s42003-022-03727-9)
Supplement: Supplementary file 1 — Supplementary Information [file 42003_2022_3727_MOESM1_ESM.pdf]

Supplementary Information

A. Data preprocessing

Here we list the preprocessing steps applied to the four types of datasets: Harry Potter (HP), SEN, FST, and Human Connectome Project (HCP). For the EEG and MEG data, please refer to this website for the details of preprocessing: `https://figshare.com/articles/dataset/MEG_EEG_data_viewing_scene_pictures/16766938`. A summary is listed in Table 1. For all datasets, we used an order 8 Chebyshev type I anti-aliasing filter in Python Scipy package[1] for downsampling. For any within-session identification task, data was z-scored within its corresponding type of dataset (target vs source). Some steps of preprocessing were performed using the python MNE package[2].

1- **HP/SEN**: The 306-channel Elekta Neuromag system was used for the recording. Source-space separation (SSS) along with Maxwell filtering and their temporal extension (tSSS)[3,4] were used for bad channel correction, head position correction, and electromagnetic artifacts removal. Empty room artifacts were removed. $1 \sim 150$ Hz bandpass filter and 60 & 120 Hz notch filter were used to remove line noise. Heartbeats and eyeblinks artifacts were removed with signal-space projection (SSP)[5]. The data was downsampled to 200 Hz and z-scored by channel within each individual and session.

2- **FST** (preprocessing pipeline was included in the source code): The 306-channel Elekta Neuromag system was used for the recording. Source-space separation (SSS) along with Maxwell filtering and their temporal extension (tSSS) were used for bad channel correction and electromagnetic artifacts removal. Empty room artifacts were removed. We didn't perform head position correction since there was no head position data. $1 \sim 150$ Hz Bandpass filter and 60 & 120 Hz Notch filter were used to remove line noise. Heartbeats and eyeblinks artifacts were also removed with SSP. The data was downsampled to 200 Hz and z-scored by channel within each individual and session.

3-**HCP**: Both resting and WM datasets were already preprocessed and downloaded from the HCP database: `https://www.humanconnectome.org/study/hcp-young-adult`. The details of the preprocessing pipeline[6] can be found at `https://www.humanconnectome.org/storage/app/media/documentation/s1200/HCP_S1200_Release_Reference_Manual.pdf`. MAGNES 3600 (4D Neuroimaging, San Diego, CA) system was used for the recording. For WM data, we looked at the TIM partition which corresponds to $-1.5 \sim 2.5$ s relative to the onset of the image. For both resting and WM data, the sampling frequency of the preprocessed data is 508.63 Hz, and 2 s of data were selected from each trial. This corresponds to the whole 1018 time points in the resting data and $[763 : 1780]$-th time point for the WM data (corresponding to $0 \sim 2$ s relative to the onset of the image). The 2 s data was then downsampled to 101.73 Hz. Data was z-scored by

channel within each individual and each data type (resting and WM). We looked at the 146 channels which were marked "good" among all the 77 overlapping individual between resting and WM.

**Steps**	**HP/SEN**	**FST**	**HCP**
**bad data**	corrected	corrected	removed
**head position**	corrected	not corrected	not corrected
**electromagnetic artifacts**	removed using SSS	removed using SSS	removed with bad data
**empty room artifacts**	removed	removed	removed
**band filtering**	$1 \sim 150$ Hz	$1 \sim 150$ Hz	$1.3 \sim 150$ Hz
**notch filtering**	60 & 120 Hz	60 & 120 Hz	$59 - 61\&119 - 121$ Hz
**ECG (heartbeat) artifacts**	removed with SSP	removed with SSP	removed with ICA
**EOG (eyeblink) artifacts**	removed with SSP	removed with SSP	removed with ICA
**downsampling**	200 Hz	200 Hz	101.73 Hz
**z-scoring**	by channel within individual, session	same	same
**shape of a trial** $[channels, timepoints]$	$[102, 100]$	$[102, 100]$	$[146, 204]$

Supplementary Table 1: Summary of the preprocessing stpes for HP, SEN, FST, and HCP data.

B. Statistical significance of the results

The identification and rank accuracy were averaged across subjects, identification runs, and session pairs. The reported accuracies, being so large, are both statistically and practically significant, and are nearly impossible to attribute to random chance, but accurately quantifying the uncertainty is challenging in our setup. Since featurization for each session of each subject was done before the matching, there is some weak dependence on the accuracy between subjects, session, and identification runs. This dependence makes it hard to analytically obtain a p-value for the accuracy. One numerical alternative is to permute the original recording within each session across subjects before performing matching, but this is computationally expensive as it involves loading and computing large chunks of data 1000s of times. Hence we provide below a (natural, but approximate) permutation-based method for a p-value to test the null that the match is a random guess.

Let $\mathbf{y}^i$ denote the true labels of session i. Note that $\mathbf{y}^i = [1, 2, 3, 4]^T$ for any session. The permutation test is performed as follows:

Algorithm 1: Null distribution for the identification/rank accuracy

$N_{null} \leftarrow \{\}$: samples for the null distribution
T: number of permutation runs
for $t \leftarrow 1$ **to** T **do**
    $\mathbf{y^i_t} \leftarrow \text{permute}(\mathbf{y}^i_t)$, $\forall i$
    Re-compute the average accuracy, a_t, using $\{\mathbf{y}^i_t\}_i$
    $N_{null} = N_{null} \cup \{a_t\}$
end
return N_{null}

To calculate the p-value, we simply compute $p = \frac{1}{T}\sum_{t=1}^{T} \mathbb{1}_{a \leq a_t}$, where a is the observed average accuracy of a feature across subjects, sessions, and identification runs. Algorithm 1 permutes the labels for each session independently but the permutation remains unchanged for the same source-target pair across identification runs.

We summarize the p-values for the identification and rank accuracy of three features on the FST, SEN, and HCP data using $T = 4999$ permutation runs. For all the p-values, since we have not encountered any a_t that exceeds the accuracy number, their values are simply $\frac{1}{T+1} = 0.0002$. We emphasize that even though these p-values are technically only approximate due to some weak dependence, the fact that we did not see a single permutation which achieved a higher accuracy than ours should convince even rigorous skeptics that it is nearly impossible to explain away our accuracies to chance.

Data	Feature	id acc	p-val: id	rank acc	p-val: rank
FST	**sp**	0.816	0.0002	0.936	0.0002
FST	**tp**	0.978	0.0002	0.994	0.0002
FST	**fq**	0.962	0.0002	0.991	0.0002
SEN	**sp**	0.719	0.0002	0.888	0.0002
SEN	**tp**	0.983	0.0002	0.996	0.0002
SEN	**fq**	0.991	0.0002	0.998	0.0002
HCP	**sp**	0.771	0.0002	0.974	0.0002
HCP	**tp**	0.159	0.0002	0.819	0.0002
HCP	**fq**	0.229	0.0002	0.845	0.0002

Supplementary Table 2: Statistical significance for the accuracy numbers.

C. Supplementary figures

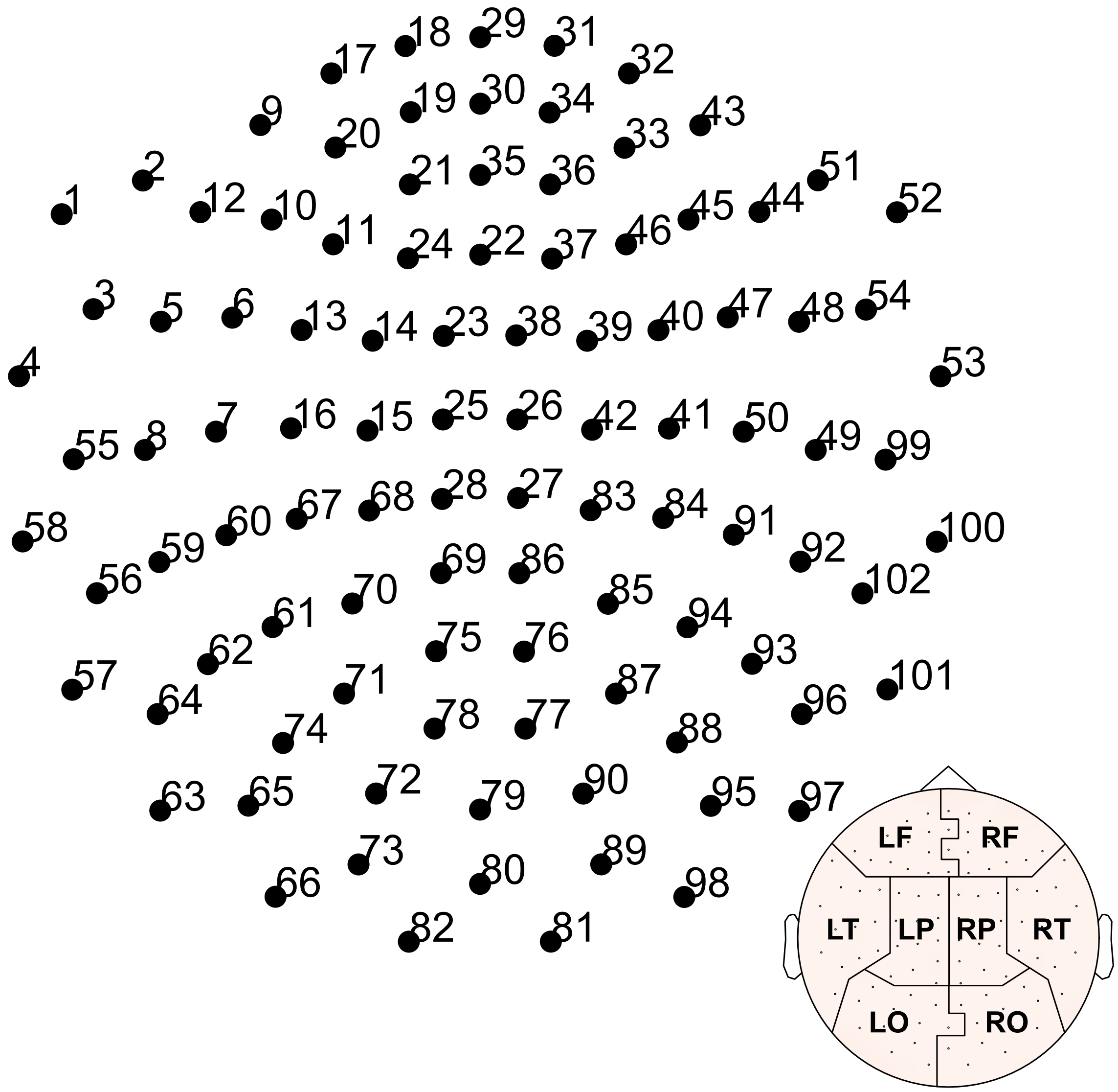

Supplementary Figure 1: Layout of the sensors for HP, FST, SEN, and MEG/EEG (MEG) data (306-channel Elekta Neuromag system). The channel numbers are consistent with the channel id of **sp** (if specified). Inset is the partitioning of the sensors same as Fig. 7(a) of the main text.

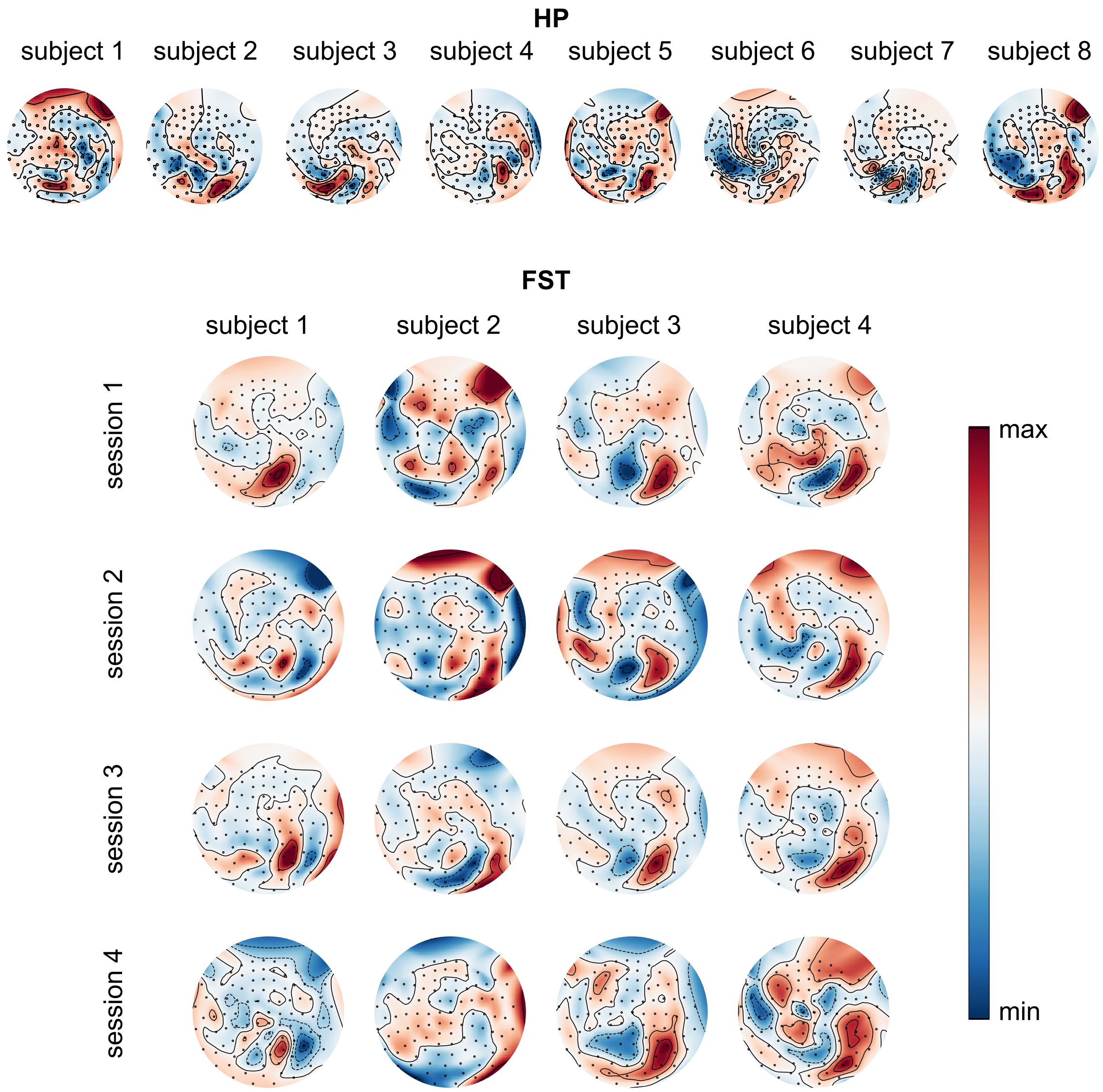

(Continued on the next page.)

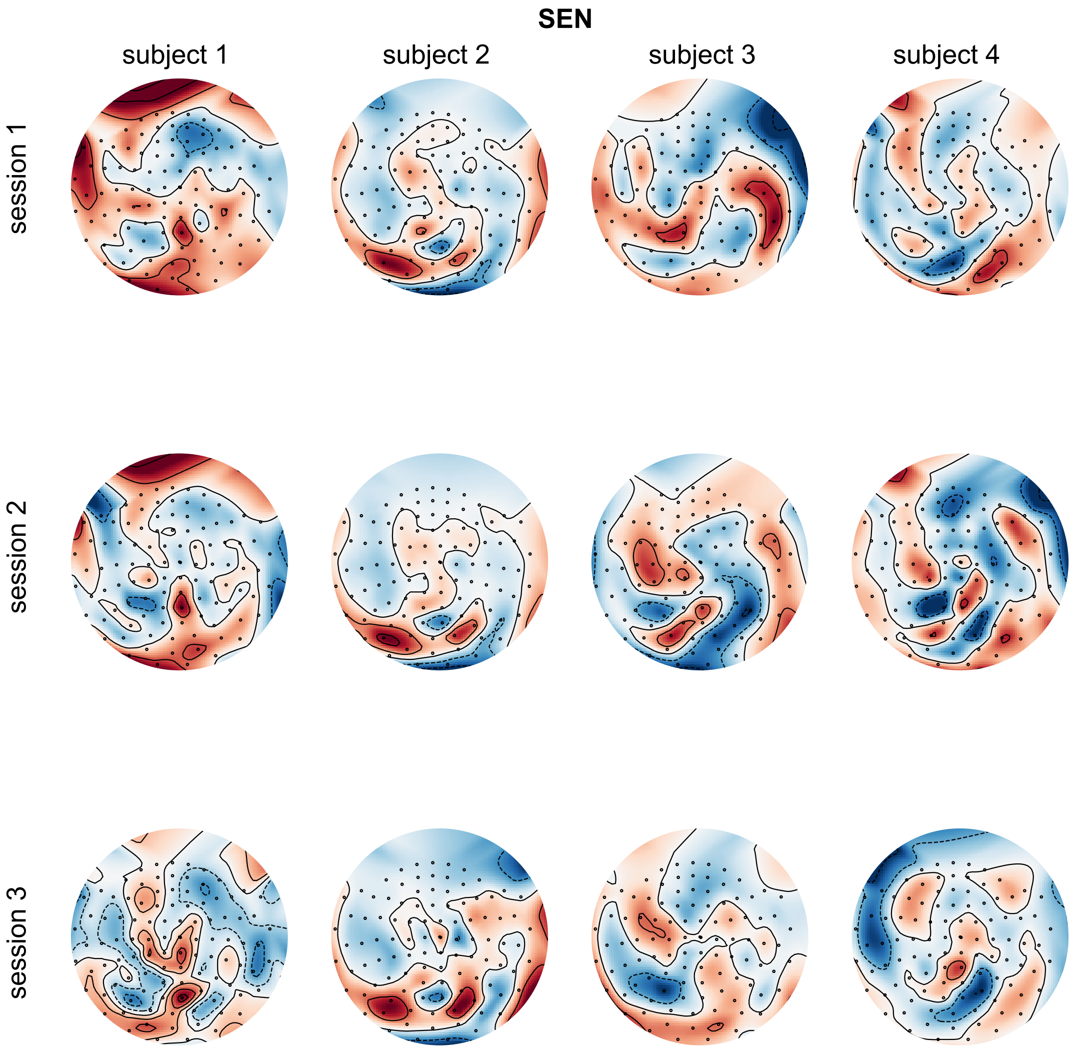

Supplementary Figure 2: Example topomaps of HP, FST, and SEN data. We plotted the topomap of these three datasets because they were all recorded using the 306-channel whole-head MEG system (Elekta Neuromag, Helsinki, Finland). Each topomap represents the spatial distribution of the brain activity at $t = 100$ ms into a trial, averaged across all trials in a session for each subject. The minimum and maximum of the colormap were specific to each topomap and for convenience, we use one colorbar to represent the scale of the color. For the multi-session FST and SEN data, the spatial distribution of the signal of an individual is more similar across sessions (within a column) as compared to that of other individuals (between columns).

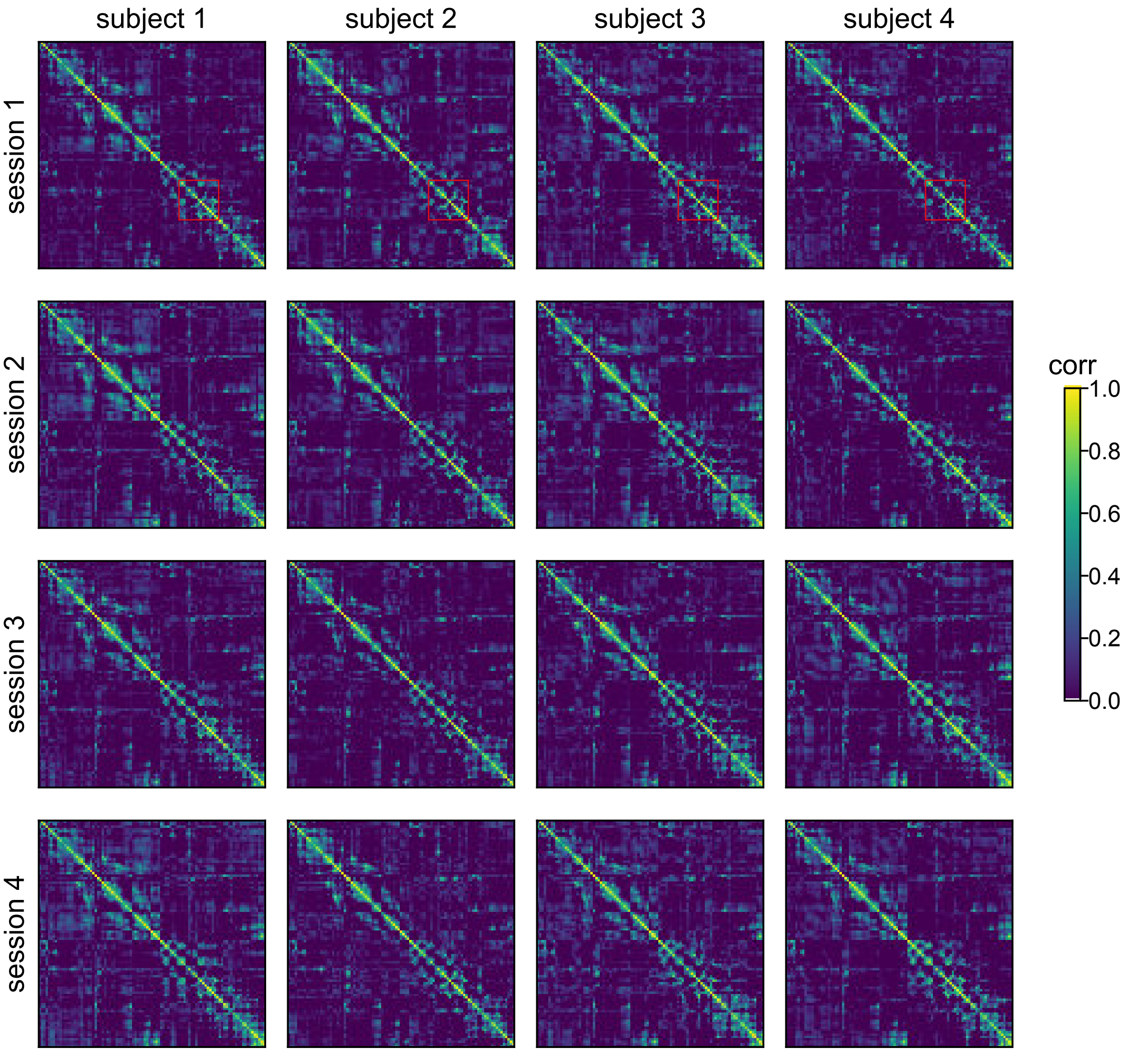

Supplementary Figure 3: Example sp (spatial connectivity) of FST data. Each heatmap represents a 102 × 102 spatial correlation matrix, where each entry represents the correlation between the brain activity on the corresponding channels. For better illustration we clipped the correlation into [0,1]. The general patterns of the correlation matrices are similar to each other. Some subsets of the heatmap, for example, the bottom-right corner, the top-left corner, and the red rectangle areas are more consistent within a individual and different between individuals. This suggests that only the interactions among a subset of sensors are individual-specific. The red rectangle areas, in particular, roughly correspond to the correlations within the left occipital (LO) lobe which yields the highest identification accuracy on both FST and SEN data (see Fig. 7(a) and Supplementary Fig. 11). New algorithms may be proposed to focus on these specific subsets to improve the identification accuracy.

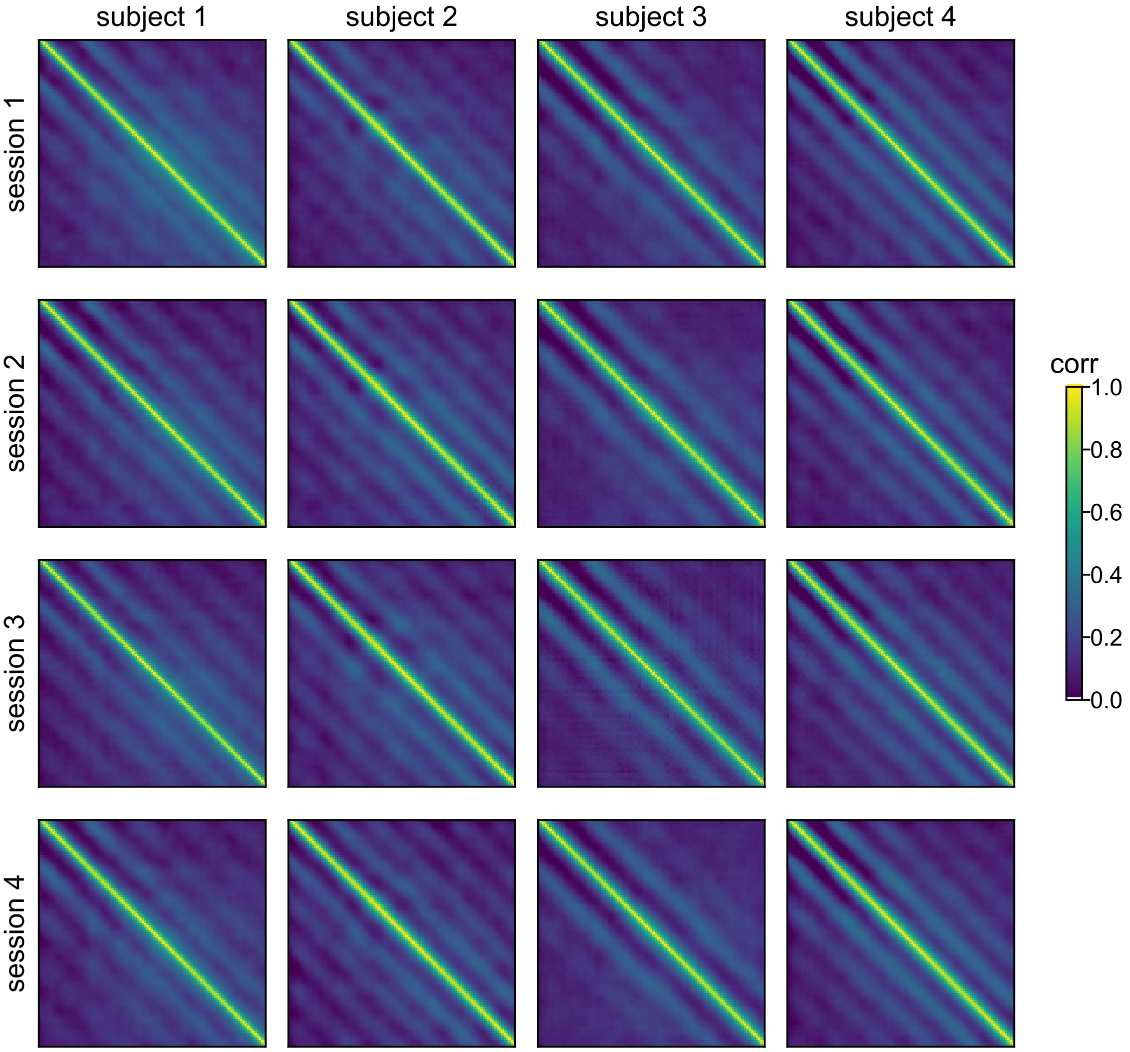

Supplementary Figure 4: Example tp (temporal connectivity) of FST data. Each heatmap represents a 100×100 temporal correlation matrix, where each entry represents the correlation between the brain activity on the corresponding time points. For better illustration we clipped the correlation into [0,1]. The banded structure of the matrices are preserved for the same individual across sessions, and are different between individuals in terms of the number of bands and the relative locations of the bands. The banded structure indicates that there are stronger correlations of the signal with itself at certain lags. In other words, looking at the auto-correlation of the signal or even cross-correlation between different channels may reveal interesting results about the temporal dynamics of the brain activities. The individual-specific band structures also confirm the findings in Fig. 7(b) that correlations of the signal with itself at certain lags are best able to identify individuals.

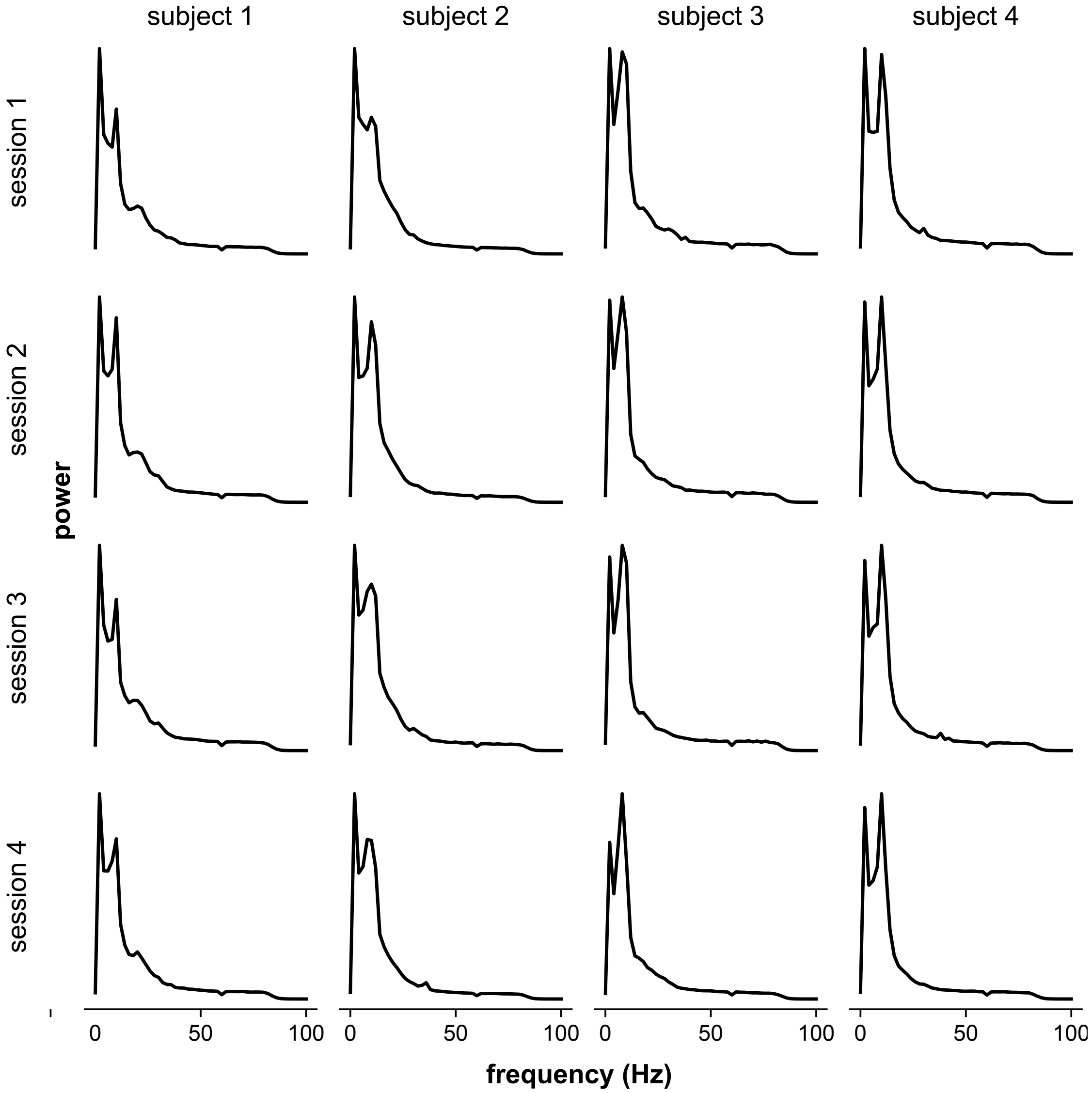

Supplementary Figure 5: Example fq (frequency) of FST data. Each plot represents power (averaged across channels) vs. frequencies (Hz), where the range of frequencies is $[0, 100]$ with a 2 Hz increment. For all individuals, there are two peaks in the power spectrum, corresponding to around 5 and 10 Hz. The relative height of the two peaks as well as the shape of the curve near the two peaks are consistently unique to an individual across sessions. There are also small peaks near 20 Hz for some individuals. These frequencies with higher amplitudes seem to align with the results shown in Fig. 7(c) where the frequency band near 10 Hz yields the highest identification accuracy. Hence the components of **fq** associated with more stimuli-driven activity or larger signal-to-noise ratio seem to yield better results.

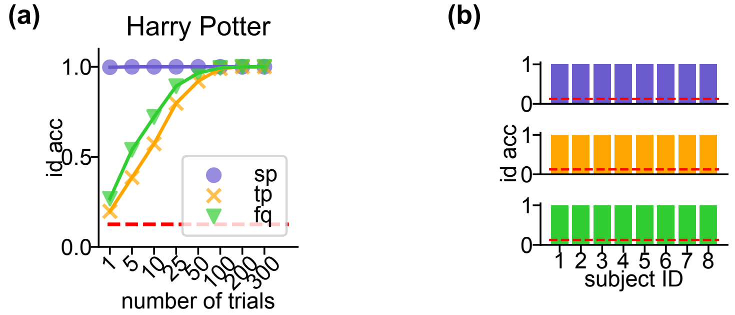

Supplementary Figure 6: Identification accuracy of sp, tp, and fq on the Harry Potter data. (a) Related to Fig. 3. Each dot was averaged across individuals (= 8) and identification runs (= 100). The red dashed line is the chance level for the identification accuracy (= $\frac{1}{8}$). Error bars are the SEs across individuals and identification runs and are invisible due to small values. Each trial is 0.5 s in length. The trends for **tp** and **fq** are similar to that of the cross-session data (SEN and FST). **sp** requires as few as one trial to achieve a perfect accuracy. This indicates strong spatial patterns in the HP data which are specific to each individual. This is expected since HP does not have more than one session, and the identification accuracy for **sp** may be lower if there are multiple sessions in HP data, similar to what we have observed on FST and SEN data. **(b)** Identification accuracy per individual for the three features on HP data using $n = 300$ trials. The red dashed lines are the chance level for the identification accuracy (= $\frac{1}{8}$). Error bars are the SEs across individuals and identification runs and are invisible due to small values.

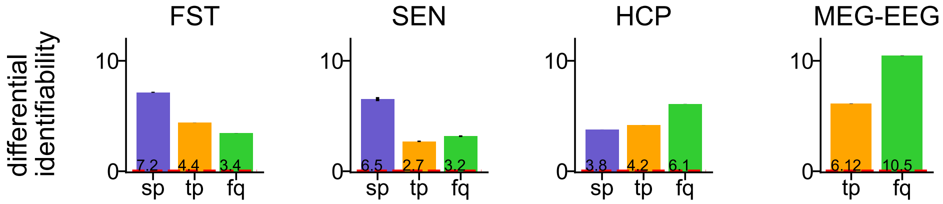

Supplementary Figure 7: Differential identifiability of the four datasets. We computed the differential identifiability of the FST, SEN, HCP, and MEG-EEG datasets. The random baseline is 0 (red dashed line). The error bars represent the SE across cross-sessions and identification runs and are invisible on some datasets. The differential identifiability for all the features considered is much larger than the random baseline on all the datasets. For FST and SEN, even though **sp** has lower identification accuracy than the other two features, their differential identifiability is still higher. This phenomenon is flipped for the HCP and MEG-EEG datasets. This might be due to the fact that many components of **tp** and **fq** are similar for the same task (i.e. in FST and SEN), leading to smaller deferential identifiability than **sp**.

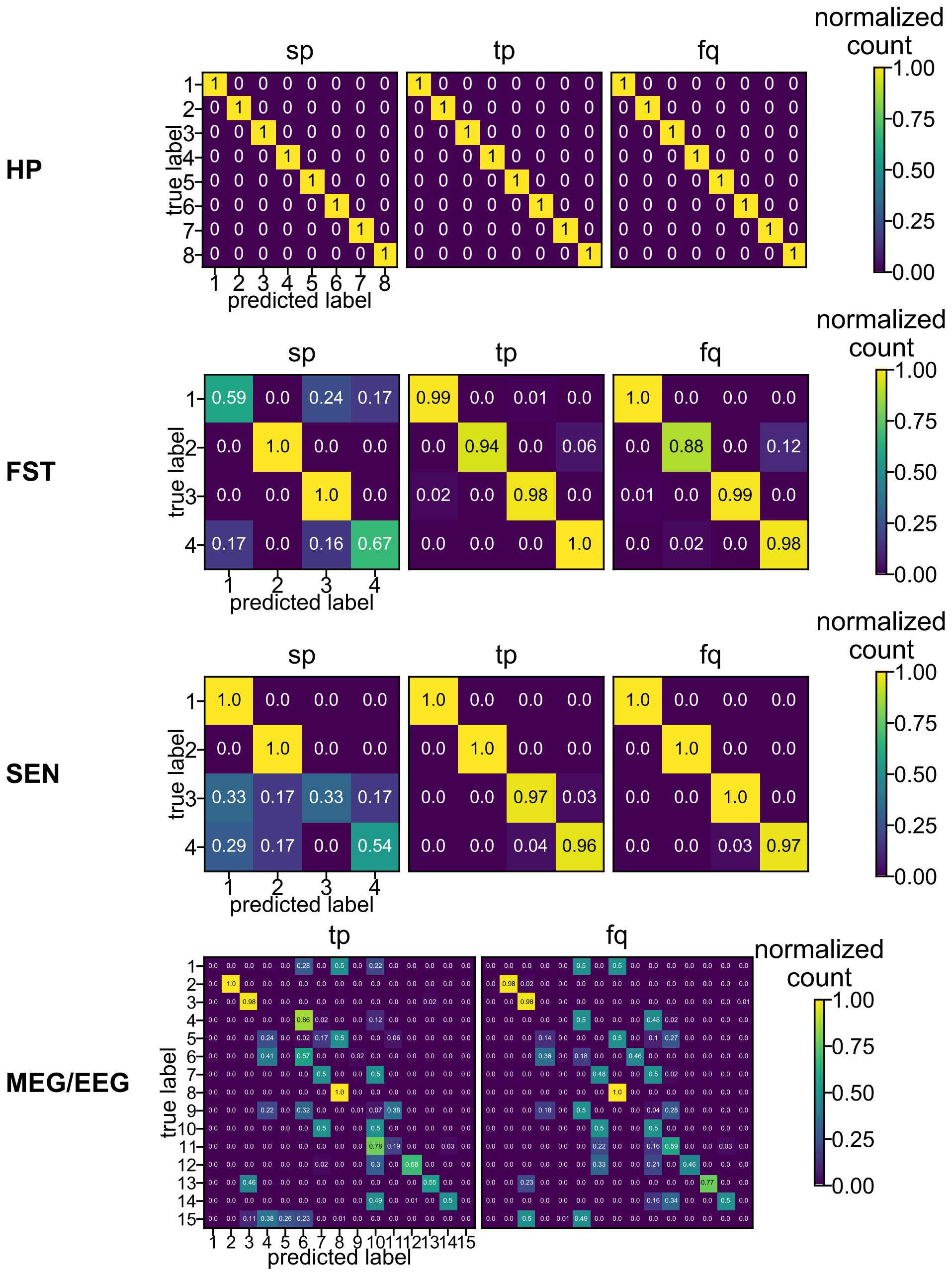

Supplementary Figure 8: Confusion matrices for HP, FST, SEN, and MEG/EEG data. For the HP, FST, SEN, and MEG/EEG datasets, we constructed the confusion matrices from the 100 identification runs of 2 cross-sessions (HP), 12 cross-sessions (FST), 6 cross-sessions (SEN), and 2 cross-modality (MEG/EEG). The numbers were normalized by the sum of each row and represent the percentage of true labels predicted as the corresponding label on the horizontal axis.

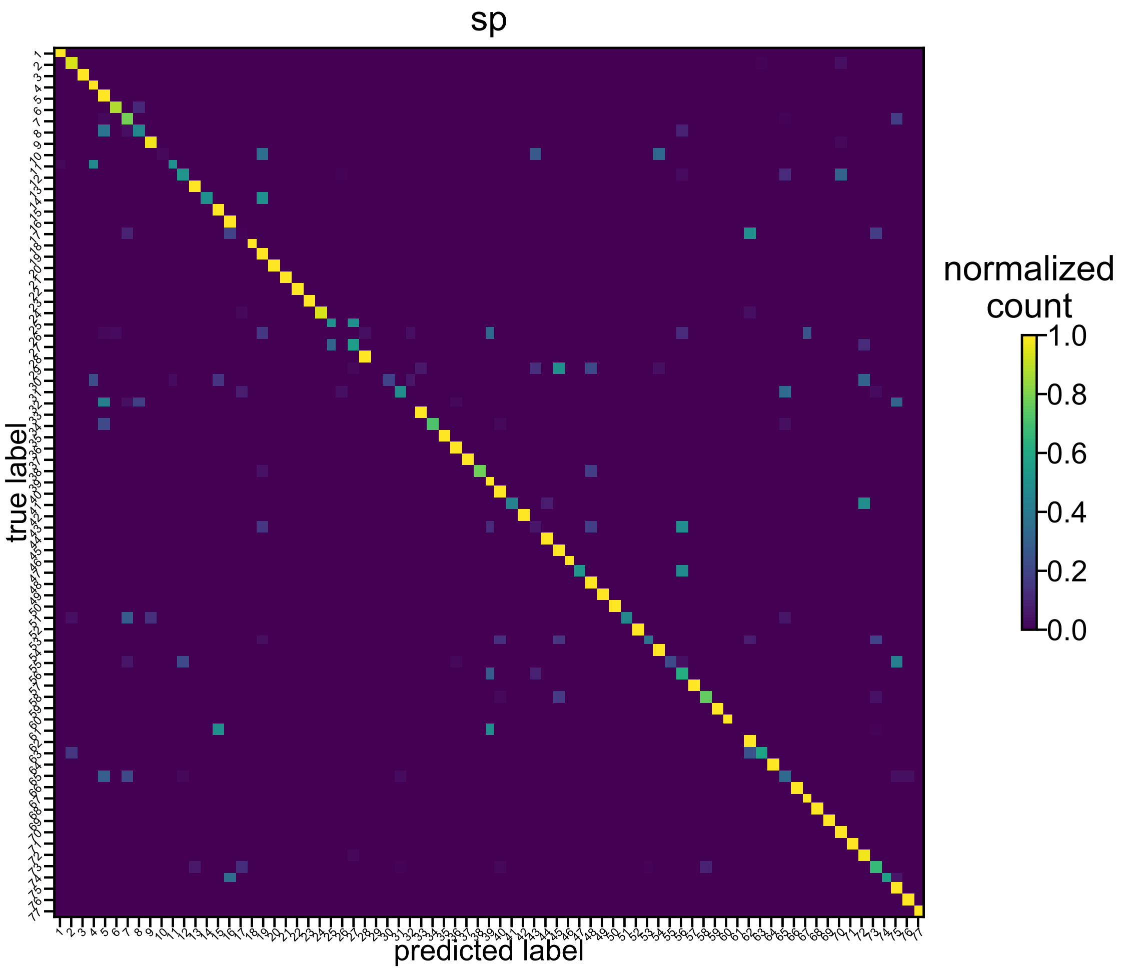

(Continued on the next page.)

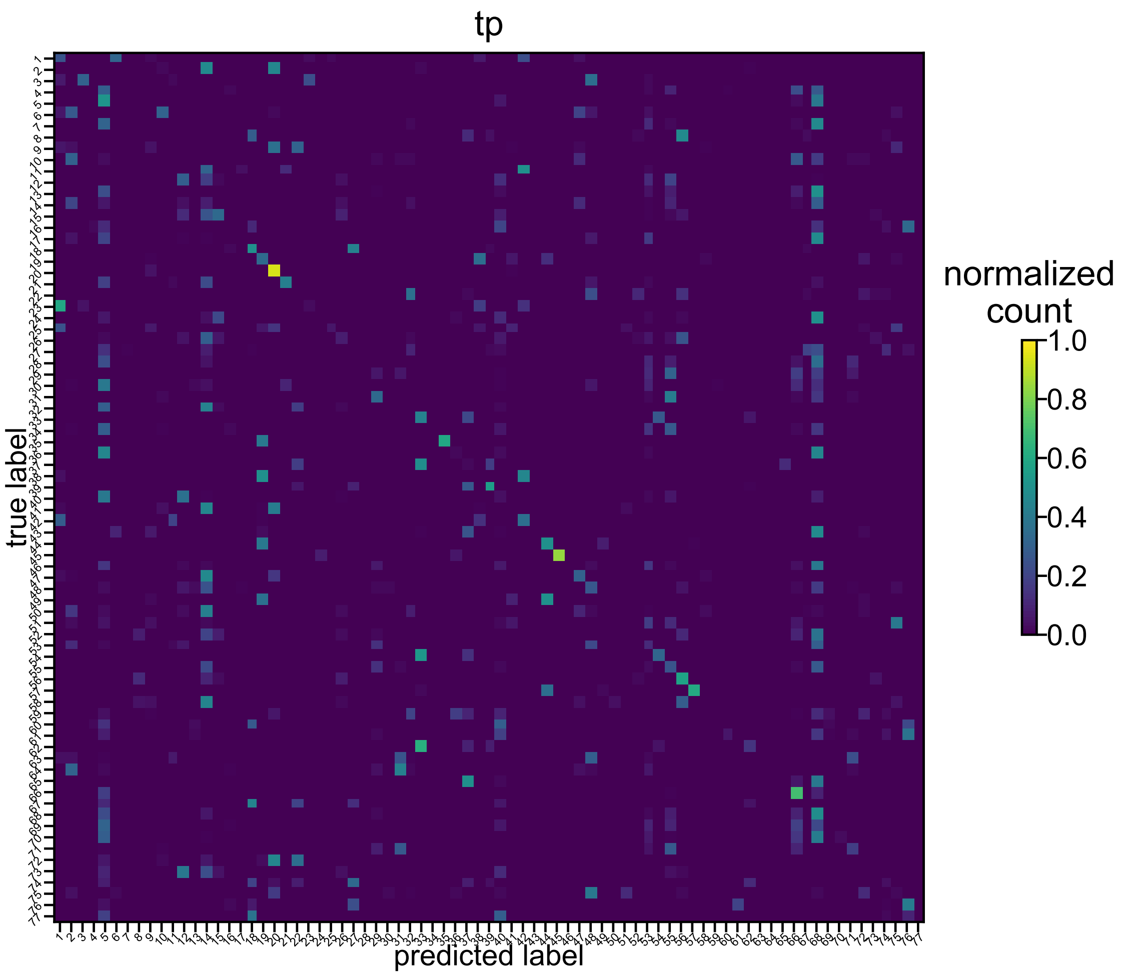

(Continued on the next page.)

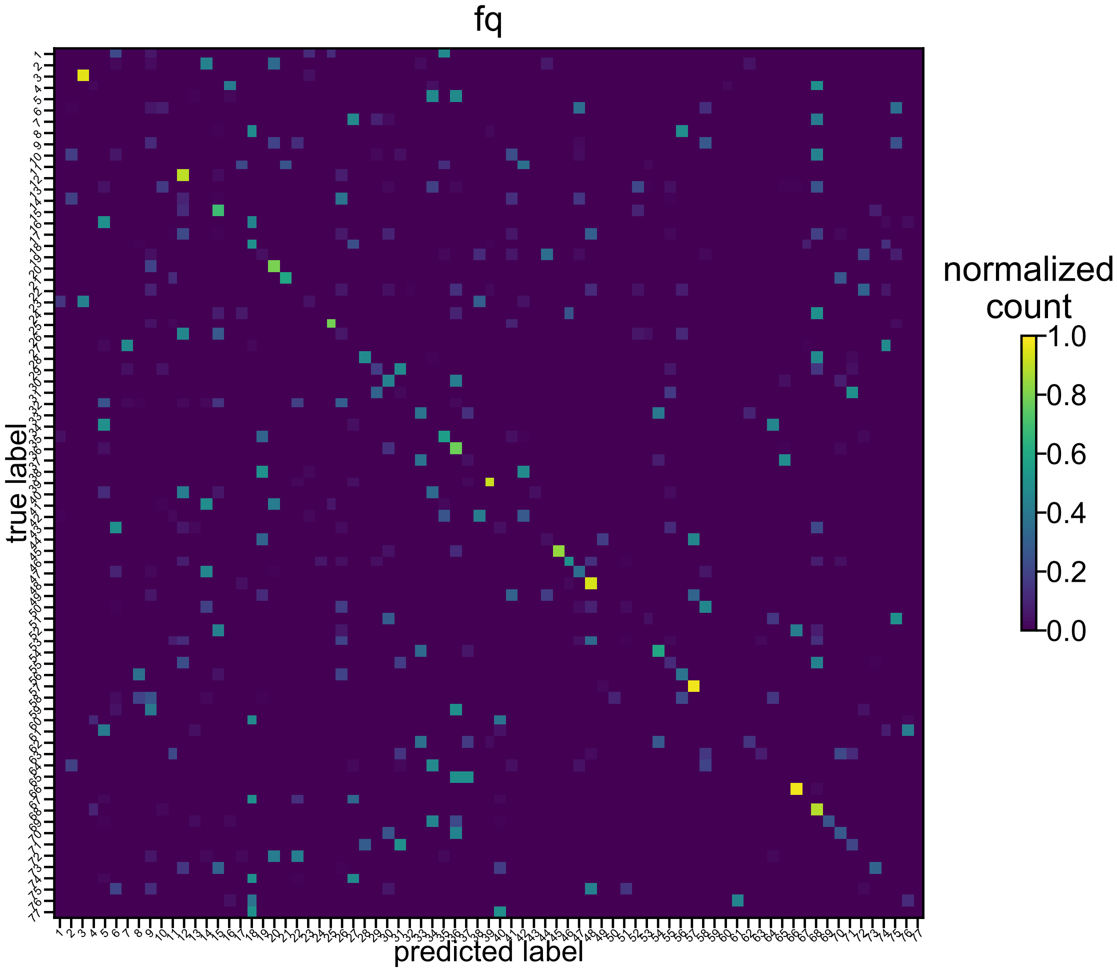

Supplementary Figure 9: Confusion matrices for HCP data. For the HCP datasets, we constructed the confusion matrices for the three features from the 100 identification runs of 2 cross-sessions. The numbers were normalized by the sum of each row and represent the percentage of true label predicted as the corresponding label on the horizontal axis.

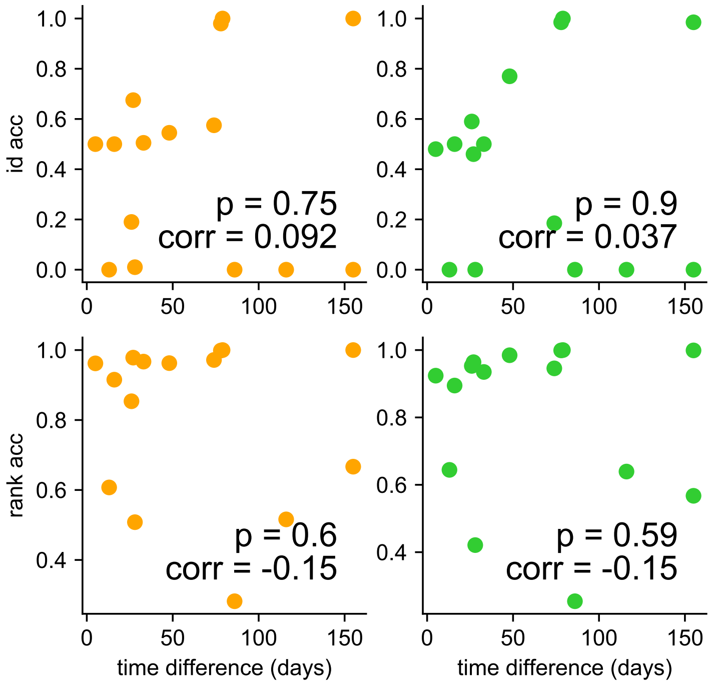

Supplementary Figure 10: Identification accuracy may not depend on the time interval between MEG and EEG data acquisition. Here we plot the identification and rank accuracy for the 15 individuals (y-axis) against the time difference between the day of MEG and EEG acquisition (x-axis, MEG data was always recorded before EEG). We computed the statistical significance at the level of individuals (Pearson's r). We do not observe a significance relationship between the accuracy and time difference. This may be due to the fact that the maximal time difference (around 150 days) may still be insufficient for identifiability to fade away. Datasets with larger range of time difference is needed to further assess the effect of time difference on individual identifiability.

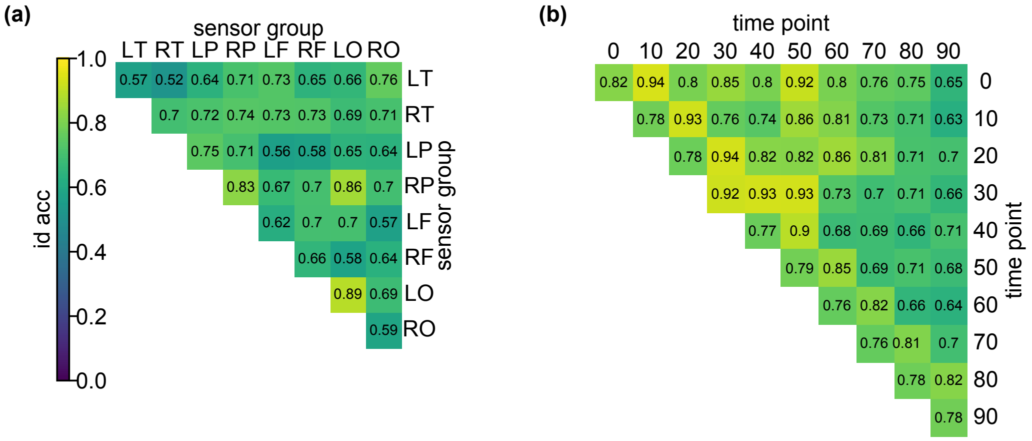

Supplementary Figure 11: Identification accuracy with sub features for a: sp and b: tp in FST data. Related to Fig. 7. For both FST and SEN, the within-LO and LO-RP correlations yield high identification accuracy. Similarly, for both FST and SEN, the super-diagonal and the correlations between the fourth and fifth 0.05 s yield high accuracy. The consistency of the results on the two datasets suggest that our conclusions regarding the importance of sub-features are not due to experiment-specific artifacts.

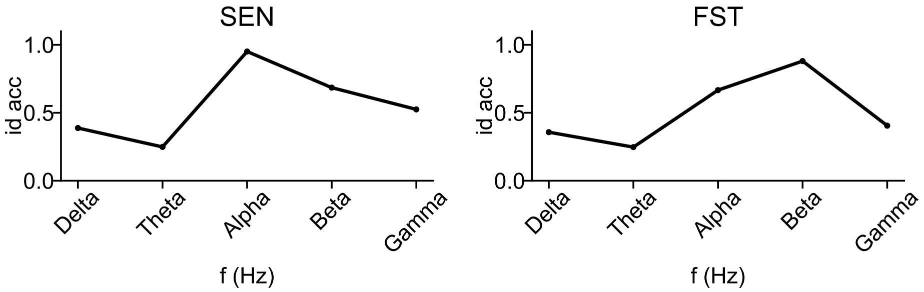

Supplementary Figure 12: Identification accuracy with respect to frequency bands. We plotted the identification accuracy with respect to different frequency bands to understand their significance on identifiability. Due to the limitation of sampling frequency, we grouped the frequencies according to this modified range: Delta ($0 \sim 4$ Hz), Theta ($4 \sim 8$ Hz), Alpha ($8 \sim 14$ Hz), Beta ($14 \sim 32$ Hz), and Gamma ($32 \sim 100$ Hz). The different peaks for SEN and FST datasets might be due to the borderline difference between Alpha and Beta bands.

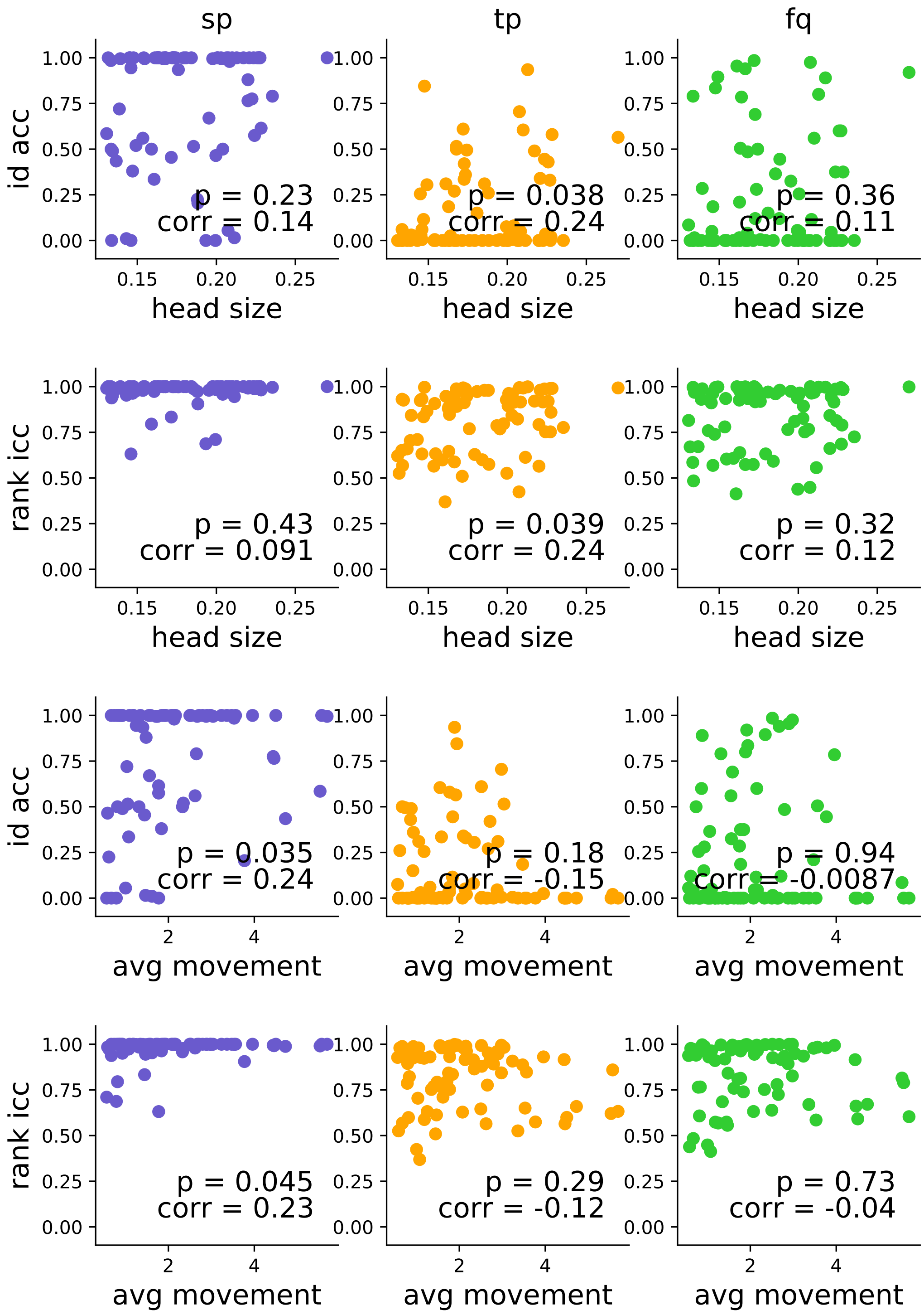

(Continued on the next page).

Supplementary Figure 13: Effect of head size and average movement on identifiability for the HCP data. For the HCP dataset, we looked at how identifiability may change with respect to the size of the individual's skull and average movement, which were both recorded for each experiment session. We plotted the identification accuracy and rank accuracy with respect to these metrics and computed the statistical significance at the level of individuals (Pearson's r). It can be seen that the average movement may have a positive impact on identification using **sp** possibly due to the fact that more movement may induce more individual-specific information. Bigger head size may lead to higher accuracy using **tp** possibly due to the more distant brain areas recorded and the more accurate average response to the stimuli. The **fq** is not affected by either metric because it is a purely spectral feature and may not be easily influenced by these spatial-related metrics. However, we do ask for caution in interpreting these results because these correlations are not statistically significant after multiple comparison corrections.

Supplementary References

[1] Pauli Virtanen, Ralf Gommers, Travis E Oliphant, Matt Haberland, Tyler Reddy, David Cournapeau, Evgeni Burovski, Pearu Peterson, Warren Weckesser, Jonathan Bright, et al. Scipy 1.0: fundamental algorithms for scientific computing in python. *Nature methods*, 17(3):261–272, 2020.

[2] Alexandre Gramfort, Martin Luessi, Eric Larson, Denis A Engemann, Daniel Strohmeier, Christian Brodbeck, Lauri Parkkonen, and Matti S Hämäläinen. Mne software for processing meg and eeg data. *Neuroimage*, 86:446–460, 2014.

[3] Samu Taulu and Matti Kajola. Presentation of electromagnetic multichannel data: the signal space separation method. *Journal of Applied Physics*, 97(12):124905, 2005.

[4] Samu Taulu and Juha Simola. Spatiotemporal signal space separation method for rejecting nearby interference in meg measurements. *Physics in Medicine & Biology*, 51(7):1759, 2006.

[5] Mikko A Uusitalo and Risto J Ilmoniemi. Signal-space projection method for separating meg or eeg into components. *Medical and Biological Engineering and Computing*, 35(2):135–140, 1997.

[6] Matthew F Glasser, Stamatios N Sotiropoulos, J Anthony Wilson, Timothy S Coalson, Bruce Fischl, Jesper L Andersson, Junqian Xu, Saad Jbabdi, Matthew Webster, Jonathan R Polimeni, et al. The minimal preprocessing pipelines for the human connectome project. *Neuroimage*, 80:105–124, 2013.
