# Supplementary material for: Brainprints: identifying individuals from magnetoencephalograms
Source: Commun Biol. 2022 Aug 22;5:852. doi: 10.1038/s42003-022-03727-9 (PMC9395342; doi:10.1038/s42003-022-03727-9)
Supplement: Supplementary file 2 — Reporting summary [file 42003_2022_3727_MOESM2_ESM.pdf]

nature portfolio

Corresponding author(s): Leila Wehbe

Last updated by author(s): May 23, 2022

Reporting Summary

Nature Portfolio wishes to improve the reproducibility of the work that we publish. This form provides structure for consistency and transparency in reporting. For further information on Nature Portfolio policies, see our Editorial Policies and the Editorial Policy Checklist.

Statistics

For all statistical analyses, confirm that the following items are present in the figure legend, table legend, main text, or Methods section.

n/a	Confirmed	
☐	☒	The exact sample size (*n*) for each experimental group/condition, given as a discrete number and unit of measurement
☐	☒	A statement on whether measurements were taken from distinct samples or whether the same sample was measured repeatedly
☐	☒	The statistical test(s) used AND whether they are one- or two-sided<br>*Only common tests should be described solely by name; describe more complex techniques in the Methods section.*
☐	☒	A description of all covariates tested
☐	☒	A description of any assumptions or corrections, such as tests of normality and adjustment for multiple comparisons
☐	☒	A full description of the statistical parameters including central tendency (e.g. means) or other basic estimates (e.g. regression coefficient) AND variation (e.g. standard deviation) or associated estimates of uncertainty (e.g. confidence intervals)
☐	☒	For null hypothesis testing, the test statistic (e.g. *F*, *t*, *r*) with confidence intervals, effect sizes, degrees of freedom and *P* value noted<br>*Give P values as exact values whenever suitable.*
☒	☐	For Bayesian analysis, information on the choice of priors and Markov chain Monte Carlo settings
☒	☐	For hierarchical and complex designs, identification of the appropriate level for tests and full reporting of outcomes
☒	☐	Estimates of effect sizes (e.g. Cohen's *d*, Pearson's *r*), indicating how they were calculated

Our web collection on statistics for biologists contains articles on many of the points above.

Software and code

Policy information about availability of computer code

Data collection	N/A
Data analysis	The code for preprocessing MEG data, computing the three features and their accuracy, and creating the figures had been uploaded to the Github repo: https://github.com/brainML/brainprint

For manuscripts utilizing custom algorithms or software that are central to the research but not yet described in published literature, software must be made available to editors and reviewers. We strongly encourage code deposition in a community repository (e.g. GitHub). See the Nature Portfolio guidelines for submitting code & software for further information.

Data

Policy information about availability of data

All manuscripts must include a data availability statement. This statement should provide the following information, where applicable:

- Accession codes, unique identifiers, or web links for publicly available datasets
- A description of any restrictions on data availability
- For clinical datasets or third party data, please ensure that the statement adheres to our policy

The HCP data can be accessed here: https://www.humanconnectome.org/study/hcp-young-adult
The FST data can be accessed here: https://figshare.com/articles/FST_raw_data/4233107
The MEG/EEG data can be accessed here: https://figshare.com/articles/dataset/MEG_EEG_data_viewing_scene_pictures/16766938
The HP and SEN data can be made available upon completing a data use agreement.

Field-specific reporting

Please select the one below that is the best fit for your research. If you are not sure, read the appropriate sections before making your selection.

☐ Life sciences ☒ Behavioural & social sciences ☐ Ecological, evolutionary & environmental sciences

For a reference copy of the document with all sections, see nature.com/documents/nr-reporting-summary-flat.pdf

Behavioural & social sciences study design

All studies must disclose on these points even when the disclosure is negative.

Study description	For all the five datasets, the FST, HCP, MEG/EEG data have their corresponding study design indicated in the cited publication (same for the sections below). For HP and SEN datasets, the data is quantitative experimental.
Research sample	The samples for HP/SEN data were collected from healthy young adults. The samples are representative for the analysis in this paper.
Sampling strategy	No sample size was determined before the study. Eight and four participants agreed to participate in the HP and SEN study.
Data collection	The data was collected using the 306-channel whole-head MEG system (Elekta Neuromag, Helsinki, Finland) at the Brain Mapping Center at the University of Pittsburgh.
Timing	N/A
Data exclusions	N/A
Non-participation	N/A
Randomization	There were no experimental/control groups.

Reporting for specific materials, systems and methods

We require information from authors about some types of materials, experimental systems and methods used in many studies. Here, indicate whether each material, system or method listed is relevant to your study. If you are not sure if a list item applies to your research, read the appropriate section before selecting a response.

Materials & experimental systems

n/a	Involved in the study	
☒	☐	Antibodies
☒	☐	Eukaryotic cell lines
☒	☐	Palaeontology and archaeology
☒	☐	Animals and other organisms
☐	☒	Human research participants
☒	☐	Clinical data
☒	☐	Dual use research of concern

Methods

n/a	Involved in the study	
☒	☐	ChIP-seq
☒	☐	Flow cytometry
☒	☐	MRI-based neuroimaging

Human research participants

Policy information about studies involving human research participants

Population characteristics	see above
Recruitment	see above
Ethics oversight	The Carnegie Mellon University and the University of Pittsburgh Institutional Review Boards have approved and overseen the Harry Potter and the SEN data collection. The other studies were accessed online and were overseen by their respective institution's Institutional Review Boards.

Note that full information on the approval of the study protocol must also be provided in the manuscript.
